# The role of ADAM8 in the mechanophenotype of MDA-MB-231 breast cancer cells in 3D extracellular matrices

**DOI:** 10.3389/fcell.2023.1148162

**Published:** 2023-05-23

**Authors:** Alexander Hayn, Tony Fischer, Claudia Tanja Mierke

**Affiliations:** Biological Physics Division, Peter Debye Institute for Soft Matter Physics, Leipzig University, Leipzig, Germany

**Keywords:** cell mechanics, extracellular matrix remodeling, fiber displacements, human breast cancer cells, collagen hydrogels, fibronectin, sheddases

## Abstract

The majority of investigations of cancer cells have been performed in an oversimplified 2D *in vitro* environment. In the last decade there is a trend toward more sophisticated 3D *in vitro* cell culture model systems that can bridge the existing gap between 2D *in vitro* and *in vivo* experiments in the field of biophysical and cell biological cancer cell research. Here, we hypothesize that the bidirectional interplay between breast cancer cells and their tumor microenvironment is critical for the outcome of the disease. Thereby, the tissue remodeling processes evoked by cancer cells are important for cancer cell-driven mechanical probing of their matrix environment and on cancer cell adhesion and motility. When remodeling processes have been explored, the emphasis was placed on matrix metalloproteinases and rather not on a disintegrin and metalloproteases (ADAMs). However, the role of ADAM8 in cell mechanics regulating cellular motility in 3D collagen matrices is still unclear. Thus, in this study, we focus on the function of ADAM8 in matrix remodeling and migration of 3D extracellular matrix scaffolds. Therefore, human MDA-MB-231 breast carcinoma cells with ADAM8 knocked down, referred to as ADAM8-KD cells, as well as MDA-MB-231 scrambled control cells, referred to as ADAM8-Ctrl cells, have been used to examine their ability to interact with and migrate in dense extracellular 3D matrices. The fiber displacements, as the capacity of cells to deform the environmental 3D matrix scaffold, has been observed. ADAM8-KD cells displace collagen fibers more strongly than ADAM8-Ctrl cells. Moreover, ADAM8-KD cells migrated more numerous in 3D collagen matrices compared to ADAM8-Ctrl cells. The impairment of ADAM8 using the ADAM8 inhibitor BK-1361 led to significantly increased fiber displacements of ADAM8-Ctrl cells to the levels of ADAM8-KD cells. In contrast, the inhibitor had no effect on ADAM8-KD cells in terms of fiber displacements as well as on the quantitative characteristics of cell invasion of ADAM8-Ctrl cells, albeit the cells that were found in the matrix invaded considerably deeper. When matrix remodeling by cells is impaired through GM6001, a broad-band metalloproteinase inhibitor, the fiber displacements of both cell types increased. In fact, ADAM8 is known to degrade fibronectin in a direct and/or indirect manner. The supplementation of fibronectin before polymerization of the 3D collagen matrices caused an enhancement in fiber displacements as well as in cell invasion into fibronectin-collagen matrices of ADAM8-Ctrl cells, whereas the fiber displacements of ADAM8-KD cells did not change. However, fibrinogen and laminin supplementation induced an increase in fiber displacements of both cell types. Thus, the impact of fibronectin on selective increase in fiber displacement of ADAM8-Ctrl cells appears to be ADAM8-dependent. As a consequence, the presence of ADAM8 may provide an explanation for the longstanding controversial results of fibronectin enrichment on malignant progression of cancers such as breast cancer. Finally, ADAM8 is apparently essential for providing cell-driven fiber displacements of the extracellular matrix microenvironment, which fosters 3D motility in a fibronectin-rich environment.

Contribution to the field. Currently, the role of ADAM8 has been explored in 2D or at maximum 2.5D in vitro cell culture motility assays. However, the mechanical characteristics of these two cell types have not been examined. In this study, the function of ADAM8 in breast cancer is refined by providing in vitro cell investigations in 3D collagen fiber matrices of various conditions. ADAM8 has been shown to be involved in the reduced generation of fiber displacements and in influencing breast cancer cell migration. However, especially in the presence of fibronectin in 3Dcollagen fiber matrices, the fiber displacements of ADAM8-Ctrl cells are increased.

## Introduction

The communication between cancer cells and their ambient extracellular matrix (ECM) and ECM-embedded cells is a mutual interplay and can impact the dynamics of tissue remodeling processes. This communication is shaped by cell-matrix interactions and ECM-remodeling through enzymes, stored substances and matrix degradation inhibiting substances that impact specific biochemical and structural characteristics (Özbek et al., 2010). The ECM defined as the matrisome can be categorized into collagens, proteoglycans and glycoproteins, including laminins, fibrinogen, elastin, fibronectin and tenascins (Naba et al., 2016). ECM elements are altered post-translationally through a large number of secreted remodeling enzymes, such as proteases. In most cancer types, beyond the cellular part, the non-cellular ECM environment in close vicinity to a solid primary tumor is severely altered in its structure, composition, stored molecules (Baghban et al., 2020) and consequently matrix mechanical characteristics (Liu et al., 2021; Watson et al., 2021).

Apart from the well-known matrix metalloproteinases (MMPs), such as Membrane type 1-matrix metalloproteinase (MT1-MMP), MMP2 and MMP9 that are linked to a poor prognosis of cancer (Têtu et al., 2006; Joseph et al., 2020, 9; Jiang and Li, 2021), even other molecules can alter the microenvironment of tumors. These molecules have been severely neglected in the past and therefore have been little or less thoroughly researched. Among them are the family of a disintegrin and metalloproteases (ADAMs) that comprise 22 members in humans, whereby ADAM8 has been even shown to cleave fibronectin (Zack et al., 2009), which is elevated in certain cancer types (Santiago-Medina and Yang, 2016; Topalovski and Brekken, 2016), such as breast cancer (Wang et al., 2017), but it is still controversially discussed (Lin et al., 2019). Also still controversially discussed is the function of fibronectin in tumorigenesis and malignant advancement (Akiyama et al., 1995; Beier et al., 2007; Lin et al., 2019). From one side, it has been published that the expression of fibronectin in cancer cells performs a tumor-suppressive function to avoid the transformation of cancers and to stop their early advancement (Taylor et al., 1998). From the other side, there is ample indication that fibronectin promotes cancer metastasis at advanced disease stages and is implicated in worse outcome when it is endogenously expressed in cancer cells. However, it remains elusive whether fibronectin impacts cells after being embedded in the ECM environment.

In general, ADAMs have been reported to be involved in proteolytic processing of membrane-bound precursors, referred to as shedding activity, and in modifying cell-cell and cell-matrix interfaces. In the last decade, the family of ADAMs has gained more and more attention and seem to include key molecules for several diseases, including cancer and its malignant progression, referred to as metastasis. In the course of metastasis, which is composed of a linear propagation of specific steps that involve the invasion of cancer cells in their ambient tumor microenvironment. The impact of ADAM8 on breast cancer cell movement in 3D environments and cell mechanics is largely less well understood. Most cancers, including breast cancers, exploit the remodeling of the ECM to establish a microenvironment that is conducive to tumorigenesis and metastasis. Moreover, cellular phenotypes and molecular functions profoundly rely on cues from sources external to the cell, such as interactions with the ECM (Mierke, 2020; Winkler et al., 2020).

Among the ADAMs only some of them are associated with malignant cancer progression. For example, there is one particularly interesting member, ADAM8, whose expression level has been associated with poor clinical prognosis in several cancers, including breast and pancreatic cancer (Schlomann et al., 2015; Conrad et al., 2018). Moreover, using transwell membrane cultures, it has been shown that ADAM8 promotes the transendothelial migration in the initial steps of the metastastatic cascade through elevation of MMP9 and shedding of PSCL-1 of the cell surface of breast cancer cells (Conrad et al., 2018). For biological function, ADAM8 requires multimerization and associates with β1 integrin on the cell surface (Zack et al., 2009). Thus, we hypothesize that ADAM8 increases the motility of breast cancer cells into 3D collagen hydrogels and alters the cellular capacity to remodel their ECM environment.

To explore the function of ADAM8 in a 3D extracellular microenvironment more comprehensively, we selected human MDA-MB-231 breast carcinoma cells in which ADAM8 is knocked down, referred to as ADAM8-KD cells and control cells that possess ADAM8, referred to as ADAM8-Ctrl cells as model systems for this study. Western blot analysis revealed that the ADAM8 knock down in MDA-MB-231 cells pronouncedly reduced the ADAM8 levels, whereas in control cells, the ADAM8 level is not affected (Conrad et al., 2018). Therefore, when using these two MDA-MB-231 cell types, differences between ADAM8 can be inferred from fiber displacements and cellular motility.

Using a 3D collagen hydrogel matrix, the fiber displacements of the two cell types were explored. As hypothesized, the two cell types exhibited different capacities to evoke fiber displacements. ADAM8-Ctrl cells displayed significantly less fiber displacements compared to ADAM8-KD cells. We found that ADAM8-KD cells and ADAM8-Ctrl cells exhibited significant differences in their invasiveness in 3D matrices. In this respect, ADAM8-KD cells manifested a higher invasiveness. Similar results were obtained when ADAM8 is impaired in ADAM8-Ctrl cells through the addition of the ADAM8 inhibitor BK-1361, resulting in an increase in fiber displacements and deeper invasion in 3D collagen matrices. In contrast, the addition of the ADAM8 inhibitor to ADAM8-KD cells did not further impair the fiber displacements. However, when using a broad-band metalloproteinase inhibitor, both cell lines displayed an elevation in fiber displacements. The addition of fibronectin prior to the polymerization of the 3D collagen matrices, led to an increase in fiber displacements as well as in invasion of ADAM8-Ctrl cells, whereas the fiber displacements of ADAM8-KD cells did not alter. However, the addition of fibrinogen and laminin did not lead to a significant difference in fiber displacements of both cell types, although the pore-sizes of all three modified collagen matrices are elevated. Thus, the effect of fibronectin on selective enhancement of ADAM8-Ctrl cells appears to be ADAM8-dependent. Consequently, the presence of ADAM8 may provide an explanation for the controversial results of fibronectin enrichment of the tumor microenvironment on the malignant progression of cancers such as breast cancer.

## Results

### ADAM8 is linked to force-driven fiber displacement during cell migration

**FIGURE 1:**
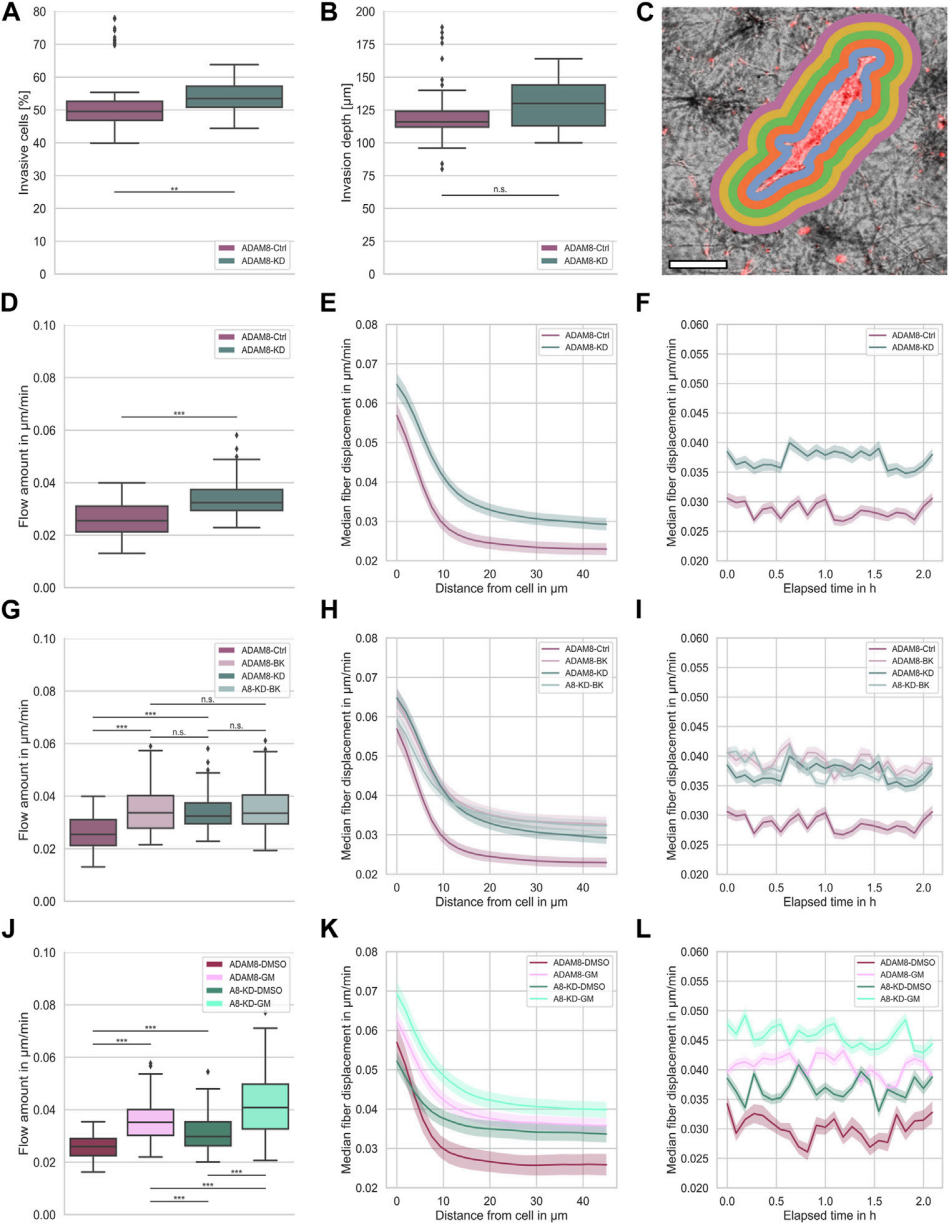
**(A)** Invasiveness of ADAM8-Ctrl (purple, n_I_ = 100, N = 6) and ADAM8-KD (cyan, n_I_ = 110, N = 6) cell lines. **(B)** Invasion depth of ADAM8-Ctrl (purple, n_I_ = 100, N = 6) and ADAM8-KD (cyan, n_I_ = 110, N = 6) cell lines. **(C)** Illustration of fiber displacement analysis. Grey is collagen matrix, red is fluorescently stained cell membrane, colorful shells represent 2D visualization of the 3D analysis of specific fiber displacement values equidistant from cell surface (each color represents different distance range). Scale bar is 20 µm. **(D)** Fiber displacements of ADAM8-Ctrl (purple, n_FD_ = 101, N = 7) and ADAM8-KD (cyan, n_FD_ = 105, N = 6) cell lines (***p < 0.001; Mann-Whitney-U-Test). **(E)** Fiber displacements of ADAM8-Ctrl (purple) and ADAM8-KD (cyan) cells in dependence of distance from cell surface. **(F)** Fiber displacements of ADAM8-Ctrl (purple) and ADAM8-KD (cyan) cells in dependence of elapsed time. **(G)** Fiber displacements of ADAM8-Ctrl (dark purple, n_FD_ = 101, N = 7) and ADAM8-KD (dark cyan, n_FD_ = 105, N = 6) cells with and without BK-1361 inhibitor treatment (with treatment displayed as light purple, named ADAM8-BK, n_FD_ = 140, N = 9, and light cyan, named A8-KD-BK, n_FD_ = 159, N = 9). **(H)** Fiber displacements in dependence of distance from cell surface. **(I)** Fiber displacements in dependence of elapsed time. **(J)** Fiber displacements of ADAM8-Ctrl cells (purple, n_FD_ = 47, N = 8) as DMSO control (named ADAM8-DMSO) and ADAM8-KD (cyan, n_FD_ = 135, N = 9) cells as DMSO control (named A8-KD-DMSO) with and without GM6001 broad spectrum MMP inhibitor treatment (with treatment displayed as bright purple, named ADAM8-GM, n_FD_ = 171, N = 9 and bright cyan, named A8-KD-GM, n_FD_ = 140, N = 8). **(K)** Fiber displacements in dependence of distance from cell surface. **(L)** Fiber displacements in dependence of elapsed time. Number of analyzed samples for fiber displacements is n_FD_, number of analyzed samples for cancer cell invasion is n_I_, N is the number of independent measurements (***p < 0.001; **p < 0.01, n. s. = not significant).

The motility of cells, such as cancer cells, has been proposed to be dependent on ADAM8, as it has been explored in transmembrane assays with Porcine Brain Endothelial Cells (PBEC) as a cell monolayer on top of the membrane and primary rat astrocytes in the upper chamber (Conrad et al., 2018). It has been revealed that ADAM8-Ctrl exhibit a greater transmigratory potential compared to ADAM8-KD cells. However, as this assay completely lacks an ECM environment, we explored the migratory capacity of the two cell types in dense 3D collagen matrices (Figures 1A,B). The ADAM8-KD cells were significantly more invasive compared to ADAM8-Ctrl cells. In particular, the number of invasive cells (Figure 1A) of ADAM8-KD cells is pronouncedly increased and the invasion depth (Figure 1B) slightly increased howbeit not significantly compared to ADAM8-Ctrl cells.

Bidirectional communication between ECM and cancer cells located in the ECM is a critical factor in carcinogenesis, and the cancer cells’ ability to migrate is crucial. In particular, cell migration depends on cues from their microenvironment and enzymatic remodeling. To shed light on the role of ADAM8 in cell migration and force generation, we investigated the fiber displacements for both ADAM8-Ctrl and ADAM8-KD human breast cancer MDA-MB-231 cell lines in dense 3.0 g/l collagen matrices.

ADAM8 is linked to matrix degradation (Cook et al., 2022). Thus, our first hypothesis was that a knock down of ADAM8 expression leads to an increase in fiber displacements. As ADAM8 is an important matrix-metallo-proteinase and highly involved in matrix degradation, we expected that the fiber displacements and consequently the force generation is lowered in the presence of ADAM8. In agreement with this, a reduction of ADAM8 expression should lead to decreased matrix degradation ability. In consequence, cells need to adapt and increase their force generation to maintain the migratory potential. In our study, we observed the generated forces of both ADAM8-Ctrl and ADAM8-KD cells by assessing their fiber displacements in dense 3.0 g/l collagen matrices (see Figure 1C for illustration). Fiber displacements were determined as described in the methods section. In fact, we observed a significant increase in fiber displacements of ADAM8-KD cells (0.034 μm/min ±0.006 μm/min) compared to ADAM8-Ctrl cells (0.026 μm/min ±0.006 μm/min), as seen in Figure 1D.

The fiber displacements are dependent on the distance from the cell. However, the increased fiber displacements of ADAM8-KD cells are higher at all distances (Figure 1E). This result indicates that the fiber displacement increase of ADAM8-KD cells is independent of the distance from the cell, as shown in Figure 1E. Additionally, we measured the fiber displacements for all observed distances dynamically over time. As can be seen in Figure 1F, the observed increase in fiber displacements of ADAM8-KD cells compared to ADAM8-Ctrl cells is independent of all time points measured. These results suggest that knocking down ADAM8 enhances force generation by augmenting fiber displacement.

To further strengthen the observed effect of ADAM8 knock down on fiber displacements, we investigated the specific effect of ADAM8 inhibition using the peptidomimetic ADAM8 inhibitor BK-1361 (Chen et al., 2016), as shown in Figures 1G–I.

More specifically, we inhibited ADAM8 in both ADAM8-Ctrl and ADAM8-KD cells in dense 3.0 g/l collagen matrices using the BK-1361 inhibitor. As shown in Figure 1G, we observed a significant increase in fiber displacements of BK-1361 treated ADAM8-Ctrl cells (referred to as ADAM8-BK cells) from 0.026 μm/min ±0.006 μm/min to 0.035 μm/min ±0.008 μm/min. More interestingly, the fiber displacements were increased to the level of the ADAM8-KD cells (0.034 μm/min ±0.006 μm/min) with no significant difference, indicating efficient and specific impairment of ADAM8 in ADAM8-KD and BK-1361 treated ADAM8-Ctrl cells. Even more intriguing, application of the ADAM8 inhibitor BK-1361 to ADAM8-KD cells (referred to as A8-KD-BK cells) did not alter fiber displacements (0.034 μm/min ±0.006 μm/min to 0.035 μm/min ±0.008 μm/min). These findings indicate that inhibition of ADAM8 with BK-1361 did not impact the fiber displacements in ADAM8-KD cells by side effects.

As mentioned above, described in the methods section and illustrated in Figure 1C, fiber displacements are dependent on the distance from the cell surface. However, the increased fiber displacements of ADAM8-BK, ADAM8-KD and A8-KD-BK cells are higher independently of distance from the cell, as shown in Figure 1H. Moreover, the fiber displacements are all at the same level. Additionally, we measured the fiber displacements for all observed distances dynamically over time. As seen in Figure 1I, the observed increase in fiber displacements is independently higher for ADAM8-KD and all inhibition conditions at all times.

These results further strengthen our hypothesis that loss of ADAM8 seems to be the main perpetrator of increased fiber displacements for both ADAM8-KD and BK-1361 treated ADAM8-Ctrl cells.

### Altered fiber displacements are matrix-metalloproteinase dependent

Since ADAM8 expression was reported to elevate the expression and/or activity of matrix-metalloproteinase 9 (MMP9) in breast cancer cells (Cook et al., 2022), it seems to be likely that MMP9 hampered the fiber displacement generation of ADAM8-Ctrl cells. To test for MMP dependencies of fiber displacements we treated ADAM8-Ctrl cells (referred to as ADAM8-GM cells) and ADAM8-KD cells (referred to as A8-KD-GM cells) with GM6001, a broad-band MMP inhibitor. To exclude DMSO based influence to the cells we additionally treated the ADAM8-Ctrl cells (referred to as ADAM8-DMSO cells) and the ADAM8-KD cells (referred to as A8-KD-DMSO cells) with DMSO as a control. As shown in Figure 1J, by treating cells with GM6001, both ADAM8-GM and A8-KD-GM cells show significantly increased fiber displacements compared to their matching DMSO control condition. Specifically, ADAM8-GM showed increased fiber displacements from 0.026 μm/min ±0.005 μm/min to 0.036 μm/min ±0.007 μm/min with GM6001 treatment. Fiber displacements of ADAM8-KD-GM cells were increased from 0.031 μm/min ±0.007 μm/min to 0.042 μm/min ±0.012 μm/min with GM6001 treatment.

Similar to BK-1361 inhibitor treatment, GM6001 treatment leads to increased fiber displacements in ADAM8-Ctrl cells, as shown in Figure 1J. Dissimilar to BK-1361 treatment, GM6001 broad-spectrum MMP inhibition in ADAM8-KD cells leads to a cell-distance (Figure 1K) and time independent (Figure 1L) increase in fiber displacements (0.042 μm/min ±0.012 μm/min), which is even higher than that of BK1361-treated ADAM8-Ctrl cells (0.035 μm/min ±0.008 μm/min) and GM6001-treated ADAM8-Ctrl cells (0.036 μm/min ±0.007 μm/min). This result indicates that the effect of GM6001 is rather not ADAM8-specific. Moreover, due to the impairment of MMP dependent matrix degradation by MMP inhibition, the fiber displacements of ADAM8-KD cells increased above the level of the fiber displacement increase of BK-1361 treated cells. This finding leads to the hypothesis that ADAM8-KD cells used a different migration mechanism than ADAM8-Ctrl cells. GM6001 inhibition forces ADAM8-KD cells to switch towards another MMP independent migration mode. Thus, a specific matrix degradation independent migration mode. In case of ADAM8-Ctrl cells these findings indicate that the ADAM8 dependent force generation is additionally dependent on ADAM8 induced MMP driven matrix degradation. The addition of GM6001 abolished this mechanism and thus elevated fiber displacements.

### ADAM8 mediated fiber displacements are elevated in collagen-fibronectin matrices

**FIGURE 2:**
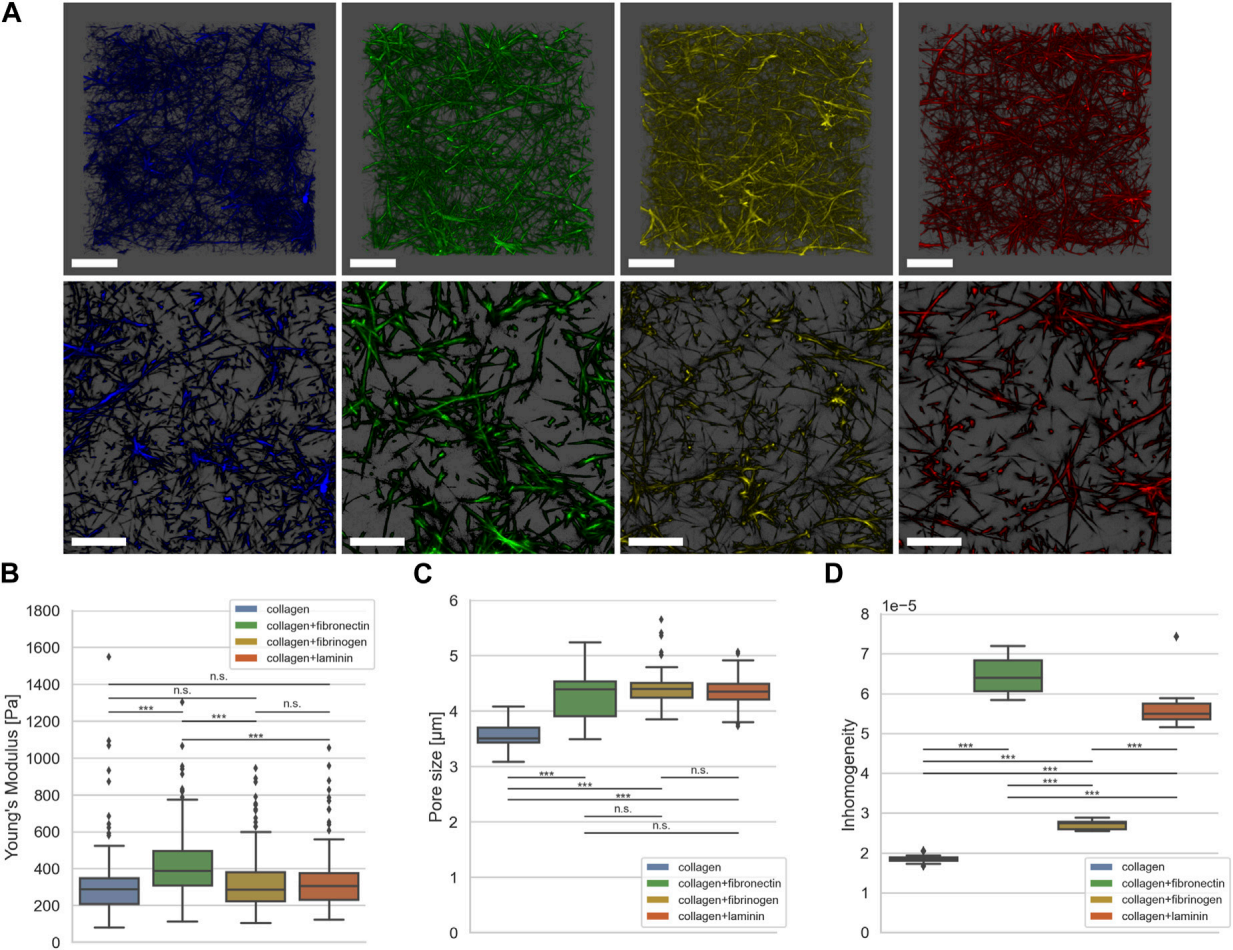
**(A)** Exemplary representative 3D LSM images of default collagen matrix (blue) and with addition of fibronectin (green), fibrinogen (yellow) and laminin A (red). **(B)** Matrix stiffness determined using AFM (collagen in blue, n_E_ = 105, N = 4, fibronectin in green, n_E_ = 175, N = 4, fibrinogen in yellow, n_E_ = 184, N = 4, laminin A in red, n_E_ = 141, N = 4). **(C)** Pore size determined using the algorithm described in (Fischer et al., 2019) (collagen in blue, n_PS_ = 65, N = 6, fibronectin in green, n_PS_ = 93, N = 8, fibrinogen in yellow, n_PS_ = 75, N = 7, laminin A in red, n_PS_ = 86, N = 8). **(D)** Inhomogeneity as described in (Hayn et al., 2020) (collagen in blue, n_P_ = 343,578, N_S_ = 15, fibronectin in green, n_P_ = 128,890, N_S_ = 15, fibrinogen in yellow, n_P_ = 257,322, N_S_ = 15, laminin A in red, n_P_ = 143,418, N_S_ = 15). Number of analyzed force-distant curves for elasticity measurements done with AFM is n_E_, number of analyzed collagen stacks for pore size is n_PS_, number of pores for determining inhomogeneity is n_P_. N is the number of independent measurements; N_S_ is the number of analyzed collagen stacks for inhomogeneity. Scale bars represent 20 µm (***p < 0.001, n. s. = not significant; Mann-Whitney-U-Test).

For the entire biological function, ADAM8 needs to multimerize and couple to β1 integrins on the cell surface. Since β1 integrins, such as α 5β1, can bind to the ECM component fibronectin, and the presence of ECM adhesion molecule fibronectin stimulates in cultured cells the secretion of MMPs, facilitating cancer cell invasion, we investigated the effect of this important ECM protein fibronectin as well as fibrinogen and laminin on fiber displacements. First, we incorporated fibronectin, fibrinogen and laminin (25 μg/ml each) into the collagen matrices and characterized their mechanical and structural parameters. The results are shown in Figure 2.

Figure 2A shows exemplary Laser Scanning Microscopic (LSM) images of dense collagen matrices without (blue) and with addition of fibronectin (green), fibrinogen (yellow) and laminin A (red). As seen in Figures 2B–D, fibronectin leads to increased matrix stiffness (429.1 Pa ±182.6 Pa), larger pore size (4.3 µm ± 0.37 µm), and increased inhomogeneity (6,44 × 10^−5^ ± 0.39×10^−5^). However, addition of fibrinogen (327.4 Pa ±156.9 Pa) or laminin A (336.4 Pa ±162.8 Pa) leads to no significant alteration of matrix stiffness compared to a pure collagen matrix (332.6 Pa ±215.3 Pa), as seen in Figure 2B. Interestingly, addition of either ECM protein leads to a significant increase in pore size for all conditions (4.3 µm ± 0.37 µm for fibronectin, 4.4 µm ± 0.30 µm for fibrinogen and 4.3 µm ± 0.28 µm for laminin A) compared to collagen matrices without additional ECM proteins (3.5 µm ± 0.23), as shown in Figure 2C. Furthermore, the increase in pore size is in the same range for all three conditions; Figure 2D shows that inhomogeneity is increased by over three-fold when adding fibronectin compared to pure collagen matrices (1.85 × 10^−5^ ± 0.09 × 10^−5^ to 6,44 × 10^−5^ ± 0.39 × 10^−5^). Addition of fibrinogen leads to a slight increase (2.71 × 10^−5^ ± 0.11 × 10^−5^), addition of laminin A increases inhomogeneity almost to that of fibronectin addition (5.62 × 10^−5^ ± 0.54 × 10^−5^).

In summary, the incorporation of additional ECM proteins alters the collagen scaffolds compared to the pure collagen matrices. We and others showed, that the addition of fibronectin leads to fiber alignments (Aermes et al., 2020; Garrison and Schwarzbauer, 2021), which increases pore size and stiffness and subsequently fosters inhomogeneity. This explains the characteristics of these matrices.

Laminin, like fibronectin, has multiple binding sites and belongs to the adhesive glycoproteins (McCarthy et al., 1985). Soberly viewed and not surprising, the structural changes of collagens crosslinked with laminin showed similar changes of pore size and homogeneity as the fibronectin-collagen networks.

Compared with pure collagen networks, fibrinogen incorporation leads to significantly altered inhomogeneity but not significantly altered stiffness, although changes in pore size are significant. In contrast, compared to fibronectin-collagen and laminin-collagen networks, the inhomogeneity change of fibrinogen-collagen networks is at a lower level. Networks containing collagen and fibrinogen are assembled in a different manner. Responsible for the small inhomogeneity change due to the incorporation of fibrinogen into collagen before polymerization is an independent self-assembly of both components and local fiber-fiber interactions (Coradin et al., 2020).

**FIGURE 3:**
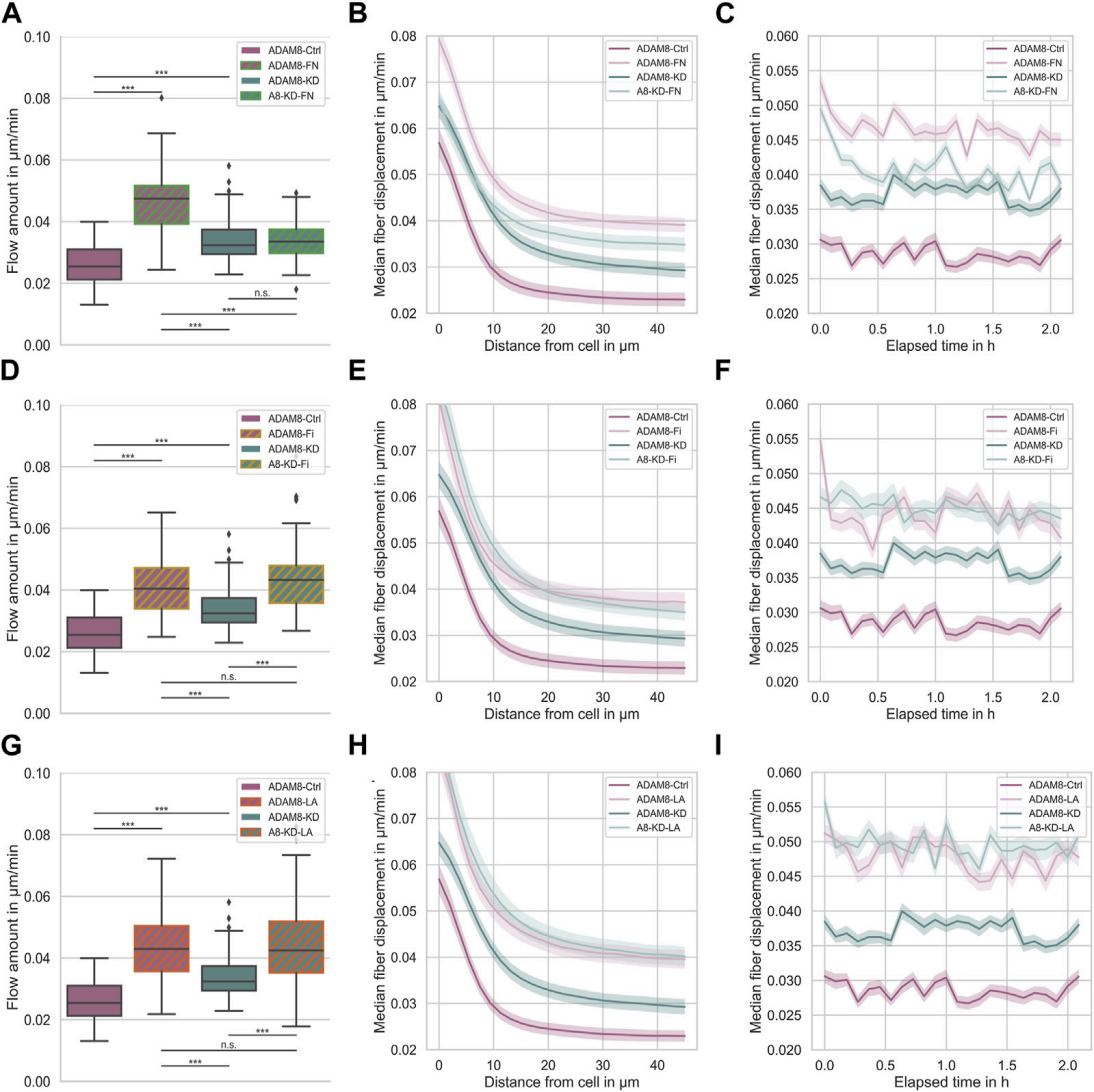
**(A)** Effect of fibronectin on fiber displacements of ADAM8-Ctrl (dark purple, n_FD_ = 101, N = 7) and ADAM8-KD (dark cyan, n_FD_ = 105, N = 6) cells in collagen matrices with and without fibronectin addition (with addition displayed as green hatches, named ADAM8-FN, n_FD_ = 84, N = 7, and named A8-KD-FN, n_FD_ = 94, N = 7). **(B)** Fiber displacements in dependence of distance from cell surface. **(C)** Fiber displacements in dependence of elapsed time. **(D)** Effect of fibrinogen on fiber displacements of ADAM8-Ctrl (dark purple, n_FD_ = 101, N = 7) and ADAM8-KD (dark cyan, n_FD_ = 105, N = 6) cells in collagen matrices with and without fibrinogen addition (with addition displayed as yellow hatches, named ADAM8-Fi, n_FD_ = 70, N = 8 and named A8-KD-Fi, n_FD_ = 70, N = 5). **(E)** Fiber displacements in dependence of distance from cell surface. **(F)** Fiber displacements in dependence of elapsed time. **(G)** Effect of laminin A on fiber displacements of ADAM8-Ctrl (dark purple, n_FD_ = 101, N = 7) and ADAM8-KD (dark cyan, n_FD_ = 105, N = 7) cells in collagen matrices with and without laminin A addition (with addition displayed as red hatches, named ADAM8-LA, n_FD_ = 84, N = 8 and named A8-KD-LA, n_FD_ = 83, N = 8). **(H)** Fiber displacements in dependence of distance from cell surface. **(I)** Fiber displacements in dependence of elapsed time. Number of analyzed samples for fiber displacements is n_FD_, N is the number of independent measurements. (***p < 0.001, n. s. = not significant; Mann-Whitney-U-Test).

Figure 3 shows the fiber displacements that ADAM8-Ctrl and ADAM8-KD cells exhibit when migrating inside the above-mentioned four different ECM model systems. According to the specific ECM protein emplacements within the 3D matrices ADAM8-Ctrl cells are referred to as ADAM8-FN when in contact with fibronectin (Figures 3A–C), ADAM8-Fi (Figures 3D–F) when in contact with fibrinogen and ADAM8-LA (Figures 3G–I) when in contact with laminin A containing collagen matrices. ADAM8-KD cells investigated within these matrices are referred to as A8-KD-FN (Figures 3A–C) for fibronectin, A8-KD-Fi (Figures 3D–F) for fibrinogen and A8-KD-LA (Figures 3G–I) for laminin A imbedding in collagen matrices. Interestingly, fiber displacements of ADAM8-Ctrl cells are increased in all three conditions, 0.047 μm/min ±0.010 μm/min for fibronectin (Figure 3A), in a cell-distance (Figure 3B) and time independent (Figure 3C) manner, 0.042 μm/min ±0.011 μm/min for fibrinogen (Figure 3D), independent from cell-distance (Figure 3E) and time (Figure 3F) and 0.045 μm/min ±0.013 μm/min for laminin A (Figure 3G), independent from cell-distance (Figure 3H) and time (Figure 3I), compared to pure collagen matrices (0.026 μm/min ±0.006 μm/min). This leads to the assumption, that the increase in fiber displacements and thus cellular force generation is caused by structural changes, such as pore size, but rather not by matrix stiffness. In addition, the elevation may also have an effect on the fiber displacements, but only a minor one. However, fiber displacements of ADAM8-KD cells are increased when adding fibrinogen (0.044 μm/min ±0.011 μm/min) (Figure 3D), independent from cell-distance (Figure 3E) and time (Figure 3F) or laminin A (0.047 μm/min ±0.016 μm/min) (Figure 3G), independent from cell-distance (Figure 3H) and time (Figure 3I) to the collagen matrix, but are not increased when adding fibronectin (0.034 μm/min ±0.006 μm/min) (Figure 3A). Fiber displacements in dependence from cell-distance (Figure 3B) are overlapping in the range of 0 μm–10 µm distance from the cell surface and are independent for distances larger than 10 µm compared to ADAM8-KD cells investigated in pure collagen matrices. Analyzed fiber displacements of A8-KD-FN cells over time (Figure 3C) were very close to each other and overlapping at some points if compared to ADAM8-KD cells in pure collagen matrices. Cell-distance and time dependencies support the consistency of the not significantly changed fiber displacement for A8-KD-FN cells compared to the fiber displacements of ADAM8-KD cells. Thus, the fact that ADAM8-KD cells show no increase of fiber displacements in fibronectin-collagen gels, but exhibit increased fiber displacements in fibrinogen-collagen and laminin A-collagen gels cannot be explained by stiffness or structure, such as increased pore-size or increased inhomogeneity. These findings lead to the hypothesis that in ADAM8-Ctrl cells ADAM8 interferes with fibronectin in a direct (shedding activity) and/or indirect manner (elevation of fibronectin degradation by MMPs).

**FIGURE 4:**
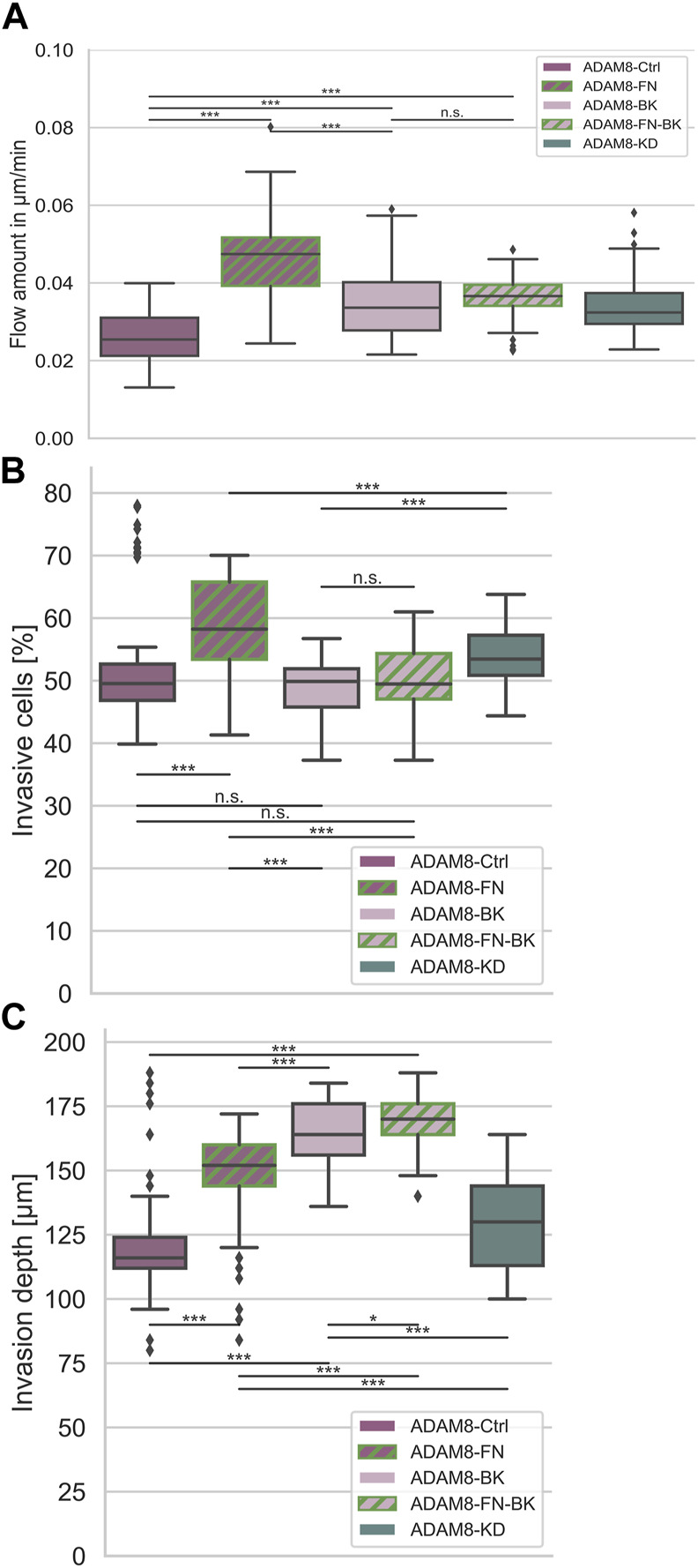
Interplay between ADAM8 and fibronectin. **(A)** Fiber displacements; ADAM8-Ctrl cells (n_FD_ = 101, N = 8) are depicted in dark purple, ADAM8 with BK inhibitor in light purple (named ADAM8-BK, n_FD_ = 133, N = 9), ADAM8-KD cells are depicted in dark cyan (n_FD_ = 105, N = 6). Addition of fibronectin to collagens is depicted as green hashes, appearing for ADAM8-Ctrl cells without BK-1361 inhibitor (dark purple with green hashes, named ADAM8-FN, n_FD_ = 81, N = 6) and with BK-1361 inhibition (light purple with green hashes, named ADAM8-FN-BK, n_FD_ = 111, N = 8). **(B)** Invasiveness and **(C)** Invasion depth of ADAM8-Ctrl cells without (purple, n_I_ = 100, N = 6) and with BK-1361 inhibition (light purple, n_I_ = 100, N = 6) and fibronectin-collagen matrices with (light purple and green hashes, n_I_ = 110, N = 6) and without BK-1361 inhibition (purple and green hashes, n_I_ = 160, N = 8) and ADAM8-KD cells (cyan, n_I_ = 110, N = 6). Number of analyzed samples for fiber displacements is n_FD_, number of analyzed samples for cancer cell invasion is n_I_, N is the number of independent measurements (***p < 0.001, *p < 0.05, n. s. = not significant).

In order to confirm that there exists a specific interplay between ADAM8 expressing cancer cells and fibronectin, we performed the fiber displacement assays in the presence and absence of the ADAM8 specific inhibitor BK-1361 using ADAM8-Ctrl and ADAM8-KD cells (Figure 4A and Supplementary Figures S1). The addition of BK-1361 to ADAM8-Ctrl cells, which have invaded in 3D fibronectin-collagen matrices (referred to as A8-FN-BK cells), abolished the fibronectin-induced elevated fiber displacements to the level of ADAM8-Ctrl cells invaded in pure collagen matrices after treatment with BK-1361 (referred to as ADAM8-BK cells), as shown in Figure 4A. Thus, the effect of fibronectin-induced elevated fiber displacement of ADAM8-Ctrl cells is abolished after addition of the BK-1361 inhibitor. In contrast and seen as a control condition, the BK-1361 inhibitor did not alter the fiber displacements of ADAM8-KD cells when invaded neither into pure collagen matrices (referred to as A8-KD-BK cells) (Supplementary Figures S1) nor into fibronectin-collagen matrices (referred to as A8-KD-FN-BK cells) (Supplementary Figures S1). This finding indicates that the effect of elevated fiber displacements of ADAM8-Ctrl cells within fibronectin-collagen matrices is ADAM8 dependent.

### Invasion depth changes due to increased fiber displacements

Invasiveness (Figure 4B) is defined as the ratio of cells invaded into a matrix to the cells that remained non-invasive. Accordingly, the invasiveness provides insight into the quantitative behavior of a large number of cells. The depth of invasion (Figure 4C) is a measure of the depth at which cells can be found after migration into the matrices and thus has quantitative and qualitative meaningfulness (Supplementary Figures S2–S5). ADAM8-Ctrl cells examined for collagen matrices with fibronectin (referred to as ADAM8-FN cells) significantly increased their invasiveness (Figure 4B) compared to the invasiveness of ADAM8-Ctrl cells and ADAM8-KD cells investigated for pure collagen matrices. The invasion depth (Figure 4C) of ADAM8-FN cells also increased significantly compared to ADAM8-Ctrl cells. Pharmacological inhibition with BK-1361 had no effect to the invasiveness (Figure 4B) of ADAM8-Ctrl cells at pure collagen matrices (referred to as ADAM8-BK cells) but increased the invasion depth of invasive cells (Figure 4C) of ADAM8-BK cells significantly compared to the ADAM8-Ctrl cells in pure collagen matrices. ADAM8-Ctrl cells that invaded collagen-fibronectin matrices treated with BK-1361 inhibitor (referred to as ADAM8-FN-BK cells) showed an invasiveness (Figure 4B) at the same level as ADAM8-Ctrl cells at pure collagen matrices and ADAM8-BK cells. The invasion depth (Figure 4C) of ADAM8-FN-BK cells is significantly higher compared to ADAM8-Ctrl cells and even higher as for ADAM8-FN, ADAM8-BK and ADAM8-KD cells. When the ADAM8-Ctrl cells come into contact with fibronectin, they increase their invasiveness. This increase is prevented by inhibition with BK-1361. This again proves an ADAM8-specific interaction with fibronectin. Especially since the invasiveness of ADAM8-FN-BK cells is similar to the invasiveness of ADAM8-BK cells. Whenever the fiber displacements increase, the depth of invasion of the cells also increases. Since the invasion depths are reached by invasive cells and the fiber displacements have been measured on invasive cells, there is a connection between these two factors. Apparently, cells are better able to migrate into the extracellular environment by interacting with it. Increased fiber displacements therefore are correlated to increased depth of invasion.

**FIGURE 5:**
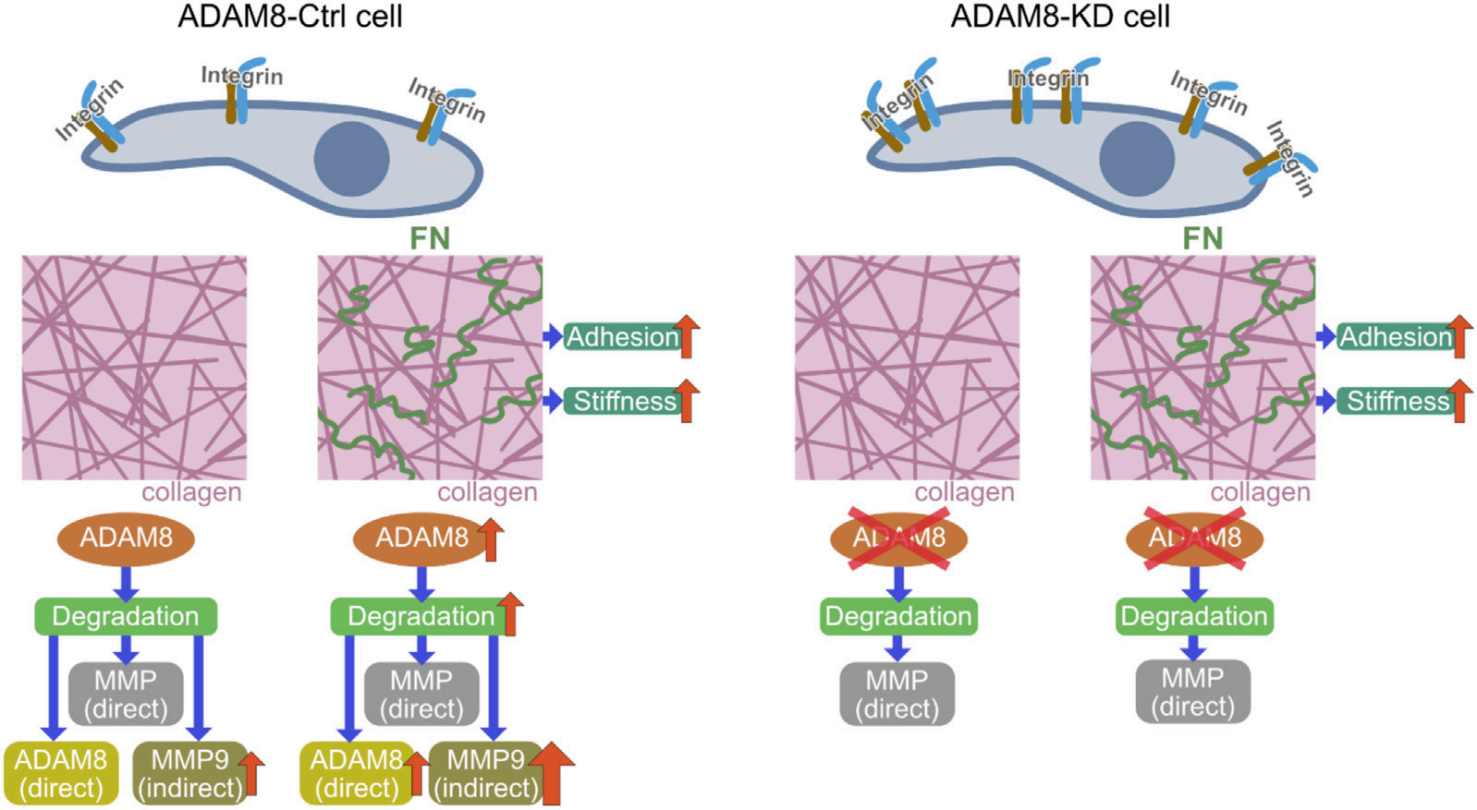
Mechanistic insight illustration of revealed potential signaling pathway; Comparison of ADAM8-Ctrl cells (left side) with ADAM8-KD cells (right side) and the ADAM8-specific effects related to the interaction of the cells with their extracellular environment. Cell-matrix interplay by integrins is influencing adhesion of cells within a collagen matrix. In the presence of fibronectin (small green squiggles in the right-hand picture of the middle row) the stiffness of the matrices increases with direct impact to the adhesion of the cells, due to integrin signaling. In case of ADAM8-Ctrl cells ADAM8 is upregulated due to the specific ADAM8-fibronectin interaction. This leads to an increased direct (ADAM8-specific) matrix-degradation and indirect degradation via MMP9 upregulation by ADAM8. ADAM8-KD that do not have ADAM8 available possess only the direct MMP-regulated degradation of the matrix. Therefore, they channel their interactions towards increased interplay with the altered matrix by fibronectin presence by increasing the deformation of the matrix by means of fiber displacements.

Finally, the results clearly show that fibronectin affects the displacement of fibers by ADAM8-Ctrl cells but has no effect on ADAM8-KD cells. Interestingly, changing the stiffness and structure (e.g., pore size and inhomogeneity) of the collagen matrix by adding other ECM proteins such as fibrinogen and laminin has an effect on the fiber displacements of both cell lines. Here, an increase in pore size can lead to better cell migration, but this is not always the case. This indicates that another mechanism seems to play a role that has a negative effect. Therefore, neither structure nor stiffness in fibronectin-collagen matrices seem to be the driving force for the increased fiber displacements of ADAM8-expressing cells, but rather an interaction between ADAM8 and fibronectin, such as a specific ADAM8 affinity for fibronectin seems to be responsible for it, as well as an indirect interaction via MMP activity increased by ADAM8. In line with this, ADAM8-Ctrl cells display reduced fiber displacements in pure collagen matrices compared to fibronectin-collagen matrices (Figure 5). This finding indicates that due to impairment of the ADAM8-specific fibronectin interaction, increased fiber displacements are not possible. Rather, an interaction between ADAM8 and fibronectin, such as a specific ADAM8 affinity for fibronectin, seems to be responsible.

## Discussion

Invasion and metastasis are the fundamental characteristics of cancer biology, represent a major hallmark of cancer and consequently, they represent the main cause of cancer mortality. With the unraveling of genetic and epigenetic mechanisms, it has come to be posited that cancer is a disorder of non-equilibrium. It is not only a disease of cancer cells, but also the misdirection of these cancer cells by the organism. The tumor microenvironment has a critical impact on cancer advancement through the co-evolution of cancer cells and cancer stroma, including the ECM scaffold of the cancer. Therefore, the investigation of the complexity of cancer propagation mechanisms from the viewpoint of tumor stroma has emerged as a new territory. The key element of the tumor stroma, the ECM, is an essential controller of cell and tissue functionalities. Traditionally, the ECM’s primary purpose has been regarded as a mere physical framework that tethers cells and tissues to one another. Nevertheless, latest research has also uncovered the biochemical and biophysical cueing characteristics of the ECM that influence cell adhesion and migration, tissue morphogenesis and reparation, as well as angiogenesis and cancer. The predominant ingredient of the ECM, collagen type I, is in charge of the primary feature of the ECM that may be linked to elevated malignancy (Pickup et al., 2014; Schwartz et al., 2017; Winkler et al., 2020).

The present study highlights the dynamic interference of ECM proteins, such as collagen type I, and breast cancer cells. Collagen type I as the major underlying structure cannot be viewed anymore as a stationary and passive support on which metastasis operates. To unravel the alterations in collagen structure and the associated cell mechanical forces that influence cancer invasion and metastasis, the decoding of the “collagen code” in carcinogenesis is an interesting area for in-depth exploration. Similar to the collagen code there may also be a “fibronectin code” that comes into play, when ADAM8 is in place. However, there seems to be neither specific “laminin code” nor “fibrinogen code” in the presence of ADAM8.

It is common knowledge that the dynamical coupling between the integrin adhesion complexes (IACs) of cells, comprising focal adhesions and invadosomes (podosomes and invadopodia), and the matrix microenvironment, is altered mainly from the cell’s cytoskeleton, cell adhesion receptors and focal adhesion proteins (Horton et al., 2016). However, the effect of shedding of cell surface receptors and the cleavage of ECM proteins is rather less well explored. While MMPs are connected to the malignant progression of several cancer types, including breast cancer, and MMPs-based cell migration has been demonstrated for 3D collagen fiber matrix assays (Zaman et al., 2006; Fraley et al., 2015; Hayn et al., 2020), the role of ADAM8 has not yet been explored in these 3D collagen fiber matrices. ADAM8 has been reported to be highly expressed in pancreas cancer (Schlomann et al., 2015; Yu et al., 2020) or ductal adenocarcinoma (Jaworek et al., 2021), is coupled to poor clinical prognosis of cancer and seems to be linked to the effect of chemoresistance to medical treatment of cancers (Conrad et al., 2019). Based on all these findings, it can be assumed that ADAM8 plays a role in cancer cell migration, such as breast cancer cell migration (Mierke, 2023). Therefore, we revealed that the motility of ADAM8 knock down MDA-MB-231 human breast cancer cells and ADAM8-Ctrl MDA-MB-231 cells in 3D collagen matrix scaffolds is altered. The ADAM8 knock down exhibited elevated invasiveness and fiber displacements compared to ADAM8-Ctrl cells, which seems to be counter-intuitive at the first glance.

Apart from structural remodeling of the ECM scaffold, the cell mechanical cues on the ECM network are important players in the malignant progression of cancers, including elevated motility via the IPP complex created by focal adhesion adaptor proteins, such as PINCH1 (Mierke et al., 2022), ILK-1 (Kunschmann et al., 2017) and Parvin. This process can promote the F-actin bundling that is a cognate process to generate a force, which can cause the displacement of ECM structures. Thus, after analyzing the fiber displacement of the two cell types in 3D collagen fiber matrices, we showed that knock down of ADAM8 increased significantly the intensity of fiber displacements possibly induced by a weakening of the connection between cell’s cytoskeleton and the extracellular microenvironment.

Preventing ADAM8 multimerization and consequently its function in MMP activation through addition of the ADAM8-specific BK-1361 inhibitor results in an elevation of fiber displacements in ADAM8-Ctrl cells. However, no additional effect can be seen in ADAM8-KD cells when treated with BK-1361. These findings indicate that the differences in fiber displacements of the two cell types are due to the presence of ADAM8. Moreover, ADAM8 needs to be multimerized to fulfill its sheddase and matrix-degrading function, which alters the behavior of cells that invaded in 3D collagen scaffolds. Therefore, the mechanisms of matrix penetration and thus cellular motility of invaded cells, may be partially impaired by ADAM8-based functions. These contribute to altering cell migration by altering fiber displacements. Thereby, ADAM8 can act as sheddase by cleaving cell adhesion receptors from the cell surface, can release substances of the ECM scaffold or degrade either direct or indirect ECM components. This first function may reduce the fiber displacement in these matrices. Similarly, the second function, such as degradation of the ECM through an ADAM8-facilitated mechanism may cause also less fiber displacements. Our results of the two cell types confirm this hypothesis, since the fiber displacements of ADAM-KD are elevated, suggesting that these cells can tether to the ECM and induce fiber displacement. This finding is further confirmed by addition of the ADAM8 inhibitor to ADAM-Ctrl cells that subsequently display elevated fiber displacement.

ADAM8 is predestined to perceive the mechanical signals from the environment and due to the molecular structure and especially its anchorage in the cell membrane it can sense and possible also store them. Another way to store mechanical information is through the cleavage of ECM proteins, such as fibronectin. In addition, laminins are critical in cancer progression (Qin et al., 2017). Moreover, also fibronectin has been reported to be crucial for malignant progression of cancers (Rick et al., 2019). Elevated plasma fibrinogen values have been noted in a variety of carcinoma types comprising breast cancer (Mei et al., 2016), gastric cancer (Zhang et al., 2016), hepatic cancer (Wang et al., 2016), and lung cancer (Liu and Shi, 2020). They have been found to be correlated with an unfavorable prognostic result of these malignancies (Tianxing et al., 2019). In addition, cancer cells are able to sequester vascular endothelial growth factor (VEGF), which continuously induces extravasation of fibrinogen (Balkwill and Mantovani, 2001). Our results on fiber displacements in collagen matrices supplemented either with fibrinogen or laminin revealed that both cell types caused similar fiber displacements in fibrinogen or laminin supplemented matrices. Thus, these fiber displacements seem to be ADAM8 independent. However, when fibronectin was supplemented to collagen matrices, the picture totally changed, as only ADAM-Ctrl cells showed elevated fiber displacements, whereas ADAM-KD cells did not. This finding indicates that there is an ADAM8 specific effect to the fiber displacements and hence force generation to fibronectin supplemented matrices. Thereby, ADAM8 can cleave fibronectin resulting in the VRAA271 neoepitope (Garcia-Pardo et al., 1983; Zack et al., 2006). However, it has been in general reported that the N-terminal part of fibronectin cross-links the ECM (Ruel et al., 2014). Moreover, MMP9 levels are reported to be induced in ADAM8-Ctrl cells and may additionally degrade fibronectin (Zack et al., 2006). In line with this, fibronectin induces MMP9 expression in ADAM8-Ctrl cells (Sen et al., 2010).

After characterizing the collagen matrices and the matrices supplemented with additional ECM proteins using AFM, the stiffnesses of the matrices (Young’s modulus) have been revealed to be at a similar level for pure collagen, laminin and fibrinogen addition. In contrast to fibronectin addition where stiffness is significantly increased compared to pure collagen matrices. More pronouncedly, the inhomogeneity increased in fibronectin supplemented collagen matrices, whereas there is also a lesser increase in laminin supplemented collagen matrices and even a weaker increase in fibrinogen supplemented collagen matrices. However, the pore sizes are elevated in all three additional supplemented collagen matrices. The pore size cannot explain the different fiber displacements in fibronectin supplemented collagen matrices of the two cell types. Rather, the increase in stiffness and inhomogeneity of fibronectin supplemented collagen matrices compared to fibrinogen and laminin, can explain the different outcomes. This finding indicates that the ADAM8-specific degradation of this collagen-fibronectin matrix may allow higher fiber displacements compared to pure collagen matrices and the other two matrices. In the lack of ADAM8, such as in ADAM8-KD cells, no further fibronectin specific impact is observed. The broad-band inhibition of matrix degrading enzymes with GM6001 increases fiber displacement in both cell types, with ADAM8-KD cells exhibiting the highest fiber displacements. The lack of specific ADAM8 dependencies means that ADAM8-KD cells have fewer opportunities to migrate and interact with the ECM. The immediate response are higher displacements. The loss of even more opportunities due to broader inhibition further limits the diversity of interactions with the matrix, possibly indicating MMP-specific migration modes of ADAM-KD cells.

ADAM8-specific influences on quantitative cell-line-specific invasion into collagen-based ECM model systems are complex and thus relatively difficult to interpret. Especially when the level of complexity of the model systems and the protagonists increases. Comparing several studies on ADAM8-induced cell invasion with the results of this study in relation to cancer cell invasion seems sobering at first glance because of the seemingly controversial results. In addition, as mentioned in the introduction, there are still many controversies about fibronectin and its role in the tumor microenvironment. Taken together, as presented in this work, the contentious tenor appears unsolvable but in case of this study they are connected like two parts of a zipper and could be teamed to support each other. Overall, this study is dealing with fiber displacements and invasion directly connected to ADAM8. While the fiber displacements provide a very specific qualitative insight into ADAM8 dependent behavior of cells that already have invaded a matrix, the results of the invasion experiments with the assay used in this study provide more quantitative results. Moreover, the invasiveness as the ratio of invaded cells to the cells that remain non-invasive is an utterly quantitative information and the second part of the invasion information, the invasion depth facilitates quantitative information of a specific group of cells, scilicet the cells that are invasive. Thus, invasion depth provides quantitative and qualitative insights. The fiber displacements, as the capacity of cells to deform the environmental 3D matrix scaffold, provide absolutely qualitative information about the behavior of invaded cells and their specific interaction with the extracellular environment. In this context, the data on the invasiveness of the cell lines used in this work do not necessarily have to correlate with the fiber displacements and therefore show different properties. The depth of invasion is partly related to the extent of fiber shifts resulting from correlation with the invading cells. This is particularly evident in the investigations of cells treated with BK-1361 inhibitor. The invasiveness of these cells remains at the level of ADAM8-Ctrl cells. This means that not more or less cells enter the matrix but the cells that are invasive are drastically influenced by this inhibition of ADAM8, as the data regarding the invasion depth reveal. Since fiber displacements of BK-1361 treated ADAM8-Ctrl cells are also increased by pharmacological inhibition, this is understandable and compatible. In case of ADAM8-Ctrl cells that are invading fibronectin-containing collagens there is an increase in invasiveness as well as in the invasion depth. Due to the direct and specific ADAM8-fibronectin connection discussed and shown by others and in this work, this circumstance is also understandable and supports all previously made statements. Based on this, and again in line with the previously determined relationships between fibronectin and ADAM8, the invasiveness of ADAM8-Ctrl cells in fibronectin-containing collagen gels decreases to the level of ADAM8-Ctrl cells when inhibited with BK-1361. This clearly shows the effect of the inhibitor and the resulting interruption of the ADAM8-fibronectin interaction during cell migration. Since fiber displacements increase in all three conditions, i.e., ADAM8-Ctrl cells in collagen matrices with fibronectin content, both with and without inhibition, and in ADAM8-Ctrl cells in pure collagen matrices with inhibition, the overall increase in the depth of invasion is also understandable. What is unusual, however, are the differences in the depth of invasion within the three conditions mentioned. The increase develops in such a way that ADAM8-Ctrl cells migrate deeper into fibronectin-containing collagen matrices compared to the cells that migrate into pure collagen gels. By inhibiting ADAM8, the cells migrate even further and finally show a further increase in the depth of invasion when both conditions, i.e., fibronectin content in the collagen gel and inhibition, meet in an almost superposition-like manner. At first glance, this seems counterintuitive. As well as the fact that the invasion behavior of cells inhibited with BK-1361 does not arrive at the level of ADAM8 knockdown cells. The reason for this is the different mode of action of pharmacological inhibition and knockdown by shRNA and the resulting different effects on cell invasion. Such differences are much discussed, including in the work of Weiss et al. (Weiss et al., 2007). This is mainly due to the fact that, in contrast to inhibition by means of shRNA, protein interactions are still possible with pharmacological suppression. Such influences, especially in quantitative approaches, such as the invasion of a high number of cells, can of course lead to different results. However, the mode of action of the inhibition itself remains unaffected, as this study shows.

If one also considers the use of different invasion methods in which other gels are used, such as Matrigel, which consist of a mixture of different ECM components, some of unknown compositions, partly with proportions of laminin, a different behavior regarding invasion compared to controlled collagen model systems is completely understandable. In particular, laminin fractions can influence the invasion, because as shown in this study, fiber displacements increase significantly due to the presence of laminin, but are ADAM8 independent. Of course, these assays are excellent for investigating specific differences with regard to invasion, because very valuable insights can be gained by directly comparing two cell lines. However, a direct comparison of the results of Matrigel invasion with invasion into controlled collagen-based systems is not always constructive. Transwell assays are often used for invasion investigations. Here, too, there are clear differences from invasion in collagen matrices. Particularly noteworthy is the use of filters with a defined pore size and the associated restriction with regard to the flexibility of the overall system. Coatings with Matrigel or even fibronectin, which has been shown to promote the invasion of ADAM8-specific cells, are also commonly used for transwell migration assays. All in all, these methods also serve in themselves as excellent systems for analyzing invasion properties. However, direct comparability with findings from differently structured systems is only given to a limited extent.

Future investigations of the nanomechanical characteristics of ADAM8 proteins will be informative. Our findings indicate that breast cancer cells preferentially lack ADAM8 to fulfill elevated migratory tasks and provide a mechanophenotype that is characterized by increased fiber displacements suggesting a force-driven migration mode. Based on these findings, we conclude that ADAM8 fulfills a crucial function in altering the mechanical phenotype of human breast cancer cells, such as reduced generation of fiber displacements. Moreover, we hypothesize that ADAM8 is also involved in sensing, possibly transducing and “coding” (storage) of mechanical cues (external inputs) through cleavage of ECM proteins, such as fibronectin.

In conclusion, our results show that ADAM8 knock down MDA-MB-231 cells were more invasive and displace the matrix environment more pronouncedly than ADAM8-Ctrl cells. These findings indicate that ADAM8 fulfills a crucial role in rather impairing MDA-MB-231 breast cancer cell locomotion. Hence, we propose a new model of the functional role of ADAM8 within the process of matrix remodeling from a biophysical viewpoint (Figure 5). These findings on ADAM8 provide a valuable resource to advance our understanding of the participation of ADAM8 in contributing to the mechanical properties of breast cancer cells and cellular motility in 3D collagen matrices and may also help to explain adverse effects in drug treatment.

## Key findings (impact on science)

• ADAM8 knock down increases fiber displacements within 3D collagen hydrogels
• ADAM8 inhibitor BK-1361 treatment increases fiber displacement of ADAM8-Ctrl cells
• ADAM8 elevates the fiber displacement within fibronectin, fibrinogen and laminin supplemented collagen matrices with highest level for fibronectin
• ADAM8 impacts the mechanophenotype of MDA-MB-231 cancer cells
• ADAM8 knock down switches the migration mode from a less matrix conversion based to a force-driven mode

## Materials and methods

### Cells and cell culture

ADAM8-Ctrl and ADAM8-KD are stable genetically modified MDA-MB-231 cell lines by transfection with shRNA as previously published by Romagnoli, et al. (Romagnoli et al., 2014) as well as by Conrad, et al. and analyzed for ADAM8 expression by ELISA, qRT-PCR and Western blotting (Conrad et al., 2018). The clones used for all experiments (namely, shCtrl-3 and shA8-20) were kindly provided by Prof. Dr. Jörg W. Bartsch (Philipps University Marburg). Cells were grown in T25 plastic cell culture flasks using 4.5 g/l DMEM cell culture medium with added 10% Fetal Bovine Serum (FBS) and 1% Penicillin/Streptomycin. Additionally, 1 μl/ml Puromycin was added as a selection antibiotic. Cells were harvested for experiments when reaching 70%–80% confluence using Trypsin/EDTA induced dislodging. Cells were incubated in an incubator at 37°C, 5% CO_2_, 95% humidity.

### 3D Collagen hydrogel preparation

Reconstituted collagen matrices were prepared as described in (Fischer et al., 2017; Hayn et al., 2020; Mierke et al., 2022). First, collagen monomers extracted from rat tail (4 mg/ml rat collagen type I, Serva, Heidelberg, Germany) and bovine skin (4 mg/ml bovine collagen type I, Biochrom, Berlin, Germany) in acidic solution at 0°C were mixed in a mass fraction of 1:2, respectively. A 1M phosphate buffered solution at 0°C was added such that the final solution has a pH value of 7.4, ionic strength of 0.7, phosphate concentration of 200 mM, and a specific monomer concentration, such as 3.0 g/l. 250 μl of this solution was added to each well of a 24-well µ-Plate (cat.no.: 82,426, ibidi, Gräfelfing, Germany) and polymerized in an incubator at 37°C, 95% humidity, for 2 h. Subsequently, the collagen scaffolds were rinsed three times with phosphate buffered saline (PBS) and stored in an incubator. Prior to any cell seeding, the polymerized collagen matrices were incubated using cell culture medium in an incubator at 37°C, 95% humidity, overnight.

### 3D Invasion assays

For invasion measurements determining invasiveness and invasion depth cells achieved in 3D collagen networks we used a well-established 3D invasion assay and collagens prepared as described in the 3D Collagen Hydrogel Preparation methods part. As described in several studies (Fischer et al., 2017; Hayn et al., 2020; Mierke et al., 2022) we used 1,200 µl solution containing collagen and buffer for each well of a 6-well plate. 3D collagen gels polymerized at 95% humidity and 37°C for 120 min. The final gels were rinsed three times with PBS before 2 ml of fresh DMEM were added to each well of the 6-well plate and incubated overnight. 50.000 harvested cells (75%–85% confluence) were added at each well on top of the collagens. Total invading time of cells was set to 72 h. In case of using pharmacological inhibitory substances, the seeded cells were allowed to settle down and adhere overnight in normal DMEM before the inhibitors were added. The 72-h invasion time was counted after addition of the inhibitors. Afterwards, we performed the fixation with 2.5% glutaraldehyde and stained the cell nuclei with the fluorophore HOECHST 33342. Image stacks were recorded using a ×20 objective, an A4 filter cube (Leica, Wetzlar, Germany) and a CCD camera (Orca-R2, Hamatsu-Photonics, Munich, Germany) mounted with a 0.55x c-mount adapter, on an inverted microscope (DMI8000B, Leica, Wetzlar, Germany). We obtained 100 image stacks, containing recordings of focal planes with a z-distance of 4 μm, received from a randomly selected 10 × 10 position grid at each well. Further, the image stacks were analyzed with a custom build python application based on elaborated algorithms for 3D image analysis and filtering. Cells found within the first two focal planes were defined as non-invasive to counterbalance minimal surface deviations. Thus, cells found 12 µm below the surface and deeper are assumed invasive.

### 3D Collagen hydrogel fiber displacement assay

For this experiment, reconstituted collagen type I matrices were prepared as described above. Cells were harvested as described above and 5,000 cells were seeded on top of each 24-well. 2 ml cell culture medium was added per well. The cells were incubated for 24 h in an incubator at 37°C, 5% CO_2_, 95% humidity. Subsequently, the samples were put in a confocal laser scanning microscope with pre-heated incubation chamber at 37°C, 5% CO_2_, 95% humidity (TCS SP8, Leica, Wetzlar, Germany). Vybrant DiD (ThermoFisher, V22887) was added to the cell medium in a concentration of 5 μl/ml and incubated for 1 h prior to each measurement. Subsequently, three-dimensional live-cell images of single cells were recorded such that the cell body and a matrix microenvironment of at least 50 µm around the cell body was present inside the 3D image. The first color channel contained the fluorescence signal of Vybrant DiD, excited by a 633 nm laser. The second color channel contained the transmitted light in a phase contrast raster scanning mode. This way, each sample contained 5-dimensional image data: a 3D image over time and two color channels containing the fluorescent cell body and surrounding ECM microenvironment.

### Modulation of fiber displacement through addition of extracellular matrix proteins

To modulate the 3D microenvironment, our well-established collagen matrix ECM model was modified by adding specific amounts of additional ECM proteins, such as fibronectin, fibrinogen and laminin A. In order to incorporate these ECM proteins into the collagen scaffolds, 25 μg/ml were added to the unpolymerized collagen solution and subsequently polymerized as described above.

### Fiber displacement analysis

To analyze the displacement of the microenvironment caused by cellular force generation during migration, an improved analysis of the already published method described in (Fischer et al., 2017; Kunschmann et al., 2019) was employed. In this study, we recorded 3D images of the fluorescently stained cell body and a 3D transmitted light image of the microenvironment. This approach is minimally invasive, as no additional markers are needed inside the collagen matrix (Steinwachs et al., 2016). The transmitted light channel shows the structure of the matrix scaffold. The recorded image data show deformations of the matrix caused by cellular forces during cell migration [Supplementary videos SV1–SV4]. This deformation was analyzed using a custom-built Python program based on optical flow analysis (Fischer et al., 2017; Kunschmann et al., 2019). The resulting data contains a tensor containing the spatial movement vectors of each 3D image pixel. In order to eliminate the movement of the cell body and do advanced deformation analysis based on distance from the cell body, further analysis was performed on the fluorescent cell body images. These images were segmented into cell body and surrounding. Subsequently, equidistant shells around the cell body were used to calculate only fiber displacements in specified distances from the cell body, as shown in Figure 1C. This way, we were able to analyze the fiber displacements over time and in dependence from distance from cell body, as shown, for example, in Figures 1D,E.

### Matrix structural and mechanical properties

Reconstituted matrices were prepared as described above. Polymerized gels were fluorescently stained using 20 μg/ml 5 (6)-Carboxytetramethylrhodamine N-succinimidylester (TAMRA-SE) (Sigma Aldrich, Cat. No: 21,955) over night, subsequently washed 3 times with PBS and kept hydrated. The 24-well µ-plate was put into a confocal laser scanning microscope (TCS SP8, Leica, Wetzlar, Germany). Using a laser wavelength of 561 nm, 3D cubic image stacks with an edge length of 100 µm were recorded. These images were analyzed as described in (Fischer et al., 2019; Hayn et al., 2020).

The elastic modulus was determined using atomic force microscopy (AFM) as published in (Hayn et al., 2020). Thus, we put 250 µl unpolymerized, cooled collagen solution into a pre-cooled Petri-dish and let this droplet polymerize at 37°C, 95% humidity, for 2 h. Subsequently, the gels were rinsed 3 times with PBS and stored hydrated at 37°C, 95% humidity in an incubator. These polymerized gels were indented using a maximum indentation force of 5 nN (Sapudom et al., 2015). We randomly picked areas along the gel surface and observed a minimum of three different regions. Each region containing at least 100 indentation points. The standard Hertz model was fitted to the retract part of the force distant curves to calculate the elastic modulus (Young’s modulus).

### Modulation of fiber displacements through pharmacological inhibitory substances

To inhibit ADAM8 expression in both ADAM8-Ctrl and ADAM8-KD cell lines, we added 500 nM of the ADAM8 specific inhibitor BK-1361. For broad spectrum MMP inhibition we added 20 µM of the broad-band MMP inhibitor GM6001. The total duration of drug treatment prior to any experiment was 24 h.

### Statistical analysis

All experimental cell invasion data were expressed as box and whiskers plots. A boxplot is a form of visualization of distributions of data based on a five-number summary, minimum and maximum, median and first and third quartiles. The top and bottom edges of the box represent the Q1 or 25th percentile and Q3 or 75th percentile, respectively. The middle line represents the median or Q2 or 50th percentile. Whiskers denote the lines that run above and below the box. The first step in determining the whiskers is to calculate the interquartile range (IQR) which is IQR = Q3-Q1. The upper and lower ends of the whiskers are usually in a distance from the box of 1.5*IQR, so Q3+1.5*IQR and Q1-1.5*IQR. The ends constitute the minimum and maximum of our data set. Any data point below or above the whisker ends is considered an outlier.

The invasion data were bootstrapped to allow hypothesis testing for otherwise singular distribution values such as depth of invasion and invasiveness of a cell population. Statistical analyses were conducted using the nonparametric Mann-Whitney U test and the Kruskal–Wallis test for non-normal distributions and unequal variances. In addition, we carried out Bonferroni corrections for each hypothesis to further enhance the statistical power of our analysis. In general, *p*-values of ≤ 0.05 were deemed statistically significant and marked with one asterisk, *p*-values of ≤ 0.01 were highlighted with two asterisks, and *p*-values of ≤ 0.001 were highlighted with three asterisks.

## Data Availability

The raw data supporting the conclusions of this article will be made available by the authors, without undue reservation.
