# Supplementary material for: The role of ADAM8 in the mechanophenotype of MDA-MB-231 breast cancer cells in 3D extracellular matrices
Source: Front Cell Dev Biol. 2023 May 23;11:1148162. doi: 10.3389/fcell.2023.1148162 (PMC10242107; doi:10.3389/fcell.2023.1148162)
Supplement: Supplementary file 3 [file DataSheet1.pdf]

The role of ADAM8 in the mechanophenotype of MDA-MB-231 breast cancer cells in 3D extracellular matrices

Alexander Hayn[⸸,⸙], Tony Fischer[⸙] and Claudia Tanja Mierke[⸙]*

Supplementary Information

Figure S1

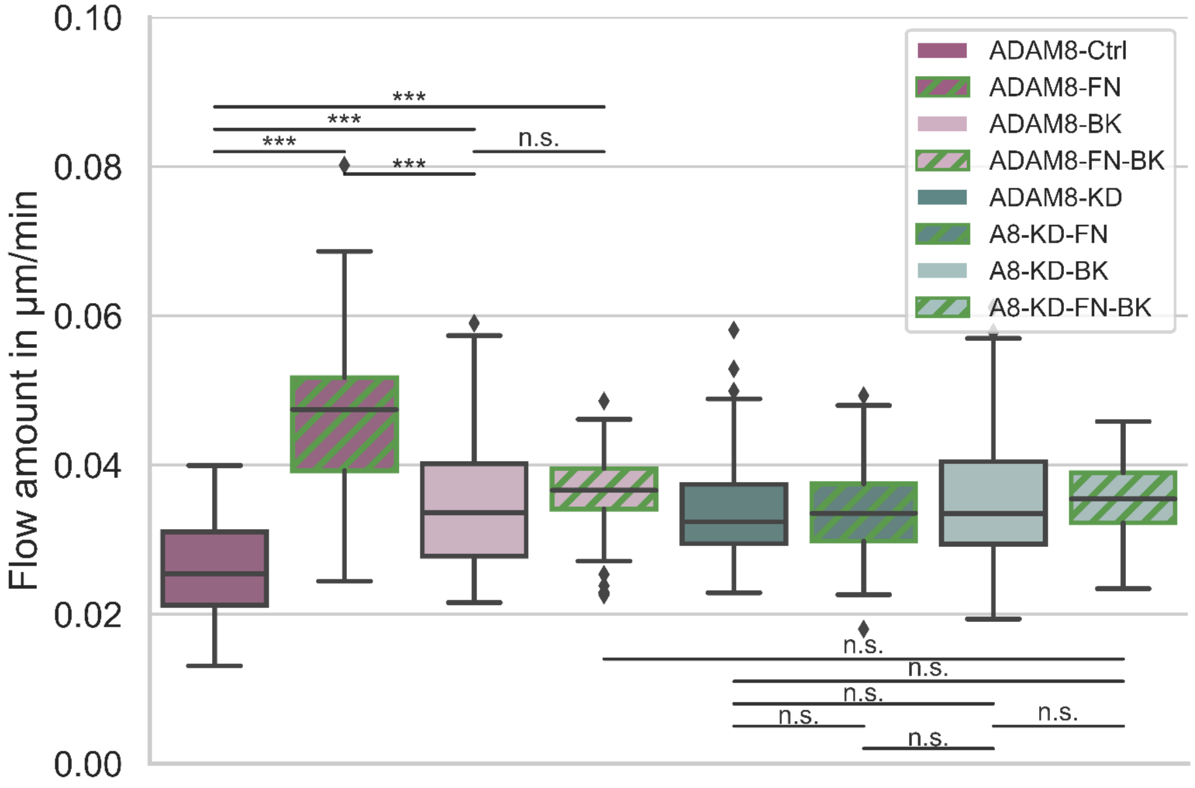

Figure S1: Interplay between ADAM8 and fibronectin. ADAM8-Ctrl cells are depicted in dark purple and ADAM8 with BK-1361 inhibitor are presented in light purple. ADAM8-KD cells are depicted in dark cyan, and ADAM8-KD with BK inhibitor are indicated with light cyan. Addition of fibronectin is depicted as green hashes.

Figure S2

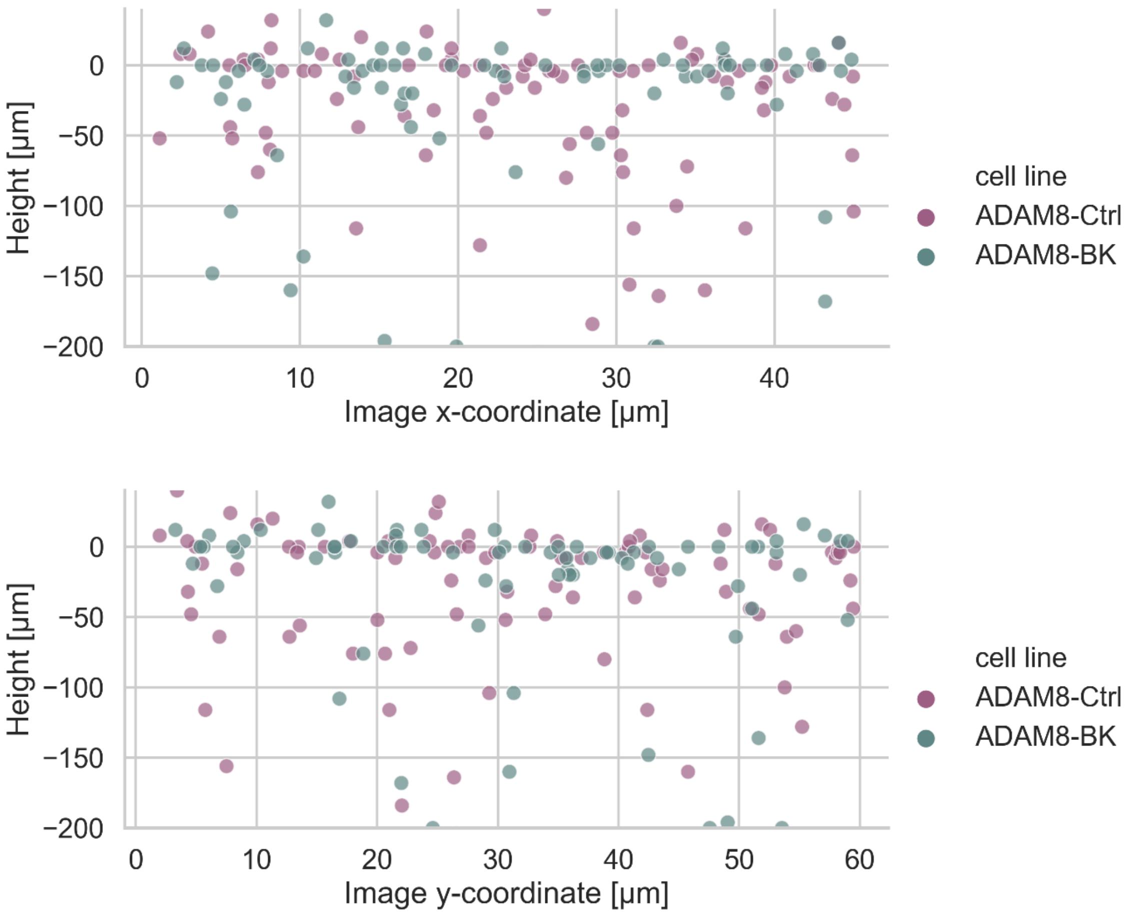

Figure S2: Exemplary comparison of the invasion of ADAM8-Ctrl cells and ADAM8-BK cells (ADAM8-Ctrl cells treated with BK-1361 inhibitor). 100 randomly selected cells from all existing stacks are shown. In each case, the x-coordinate of the determined position of the cell nuclei (upper figure) or the y-coordinate (lower figure) as a function of the depth of invasion (referred to as Height) is shown.

Figure S3

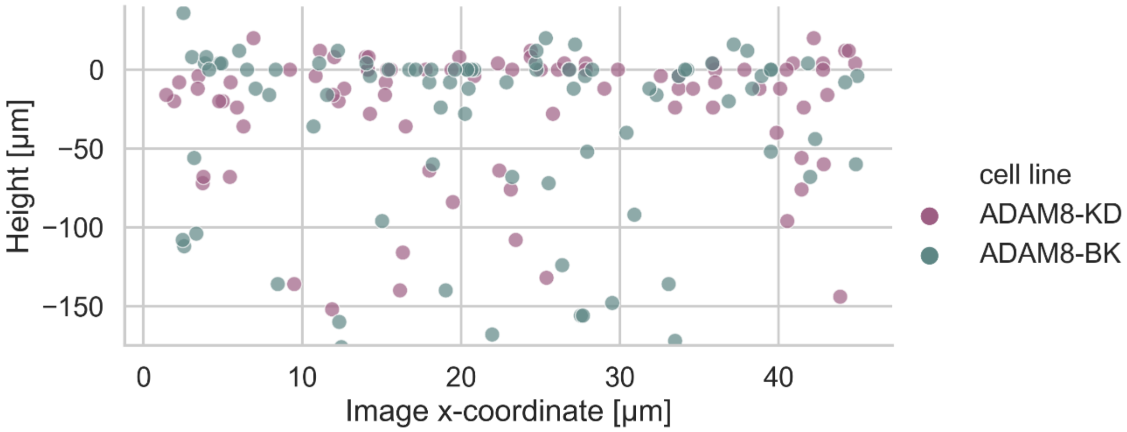

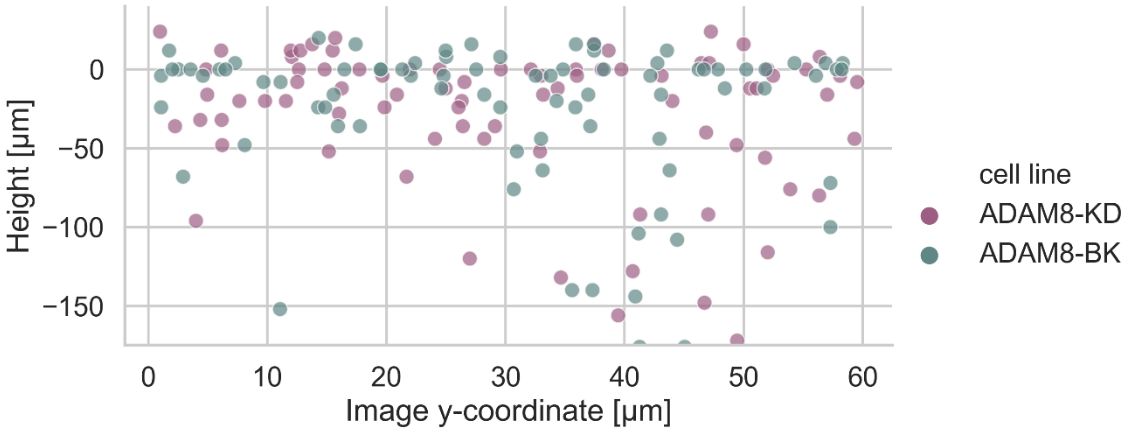

Figure S3: Exemplary comparison of the invasion of ADAM8-KD cells and ADAM8-BK cells (ADAM8-Ctrl cells under the influence of BK-1361). 100 randomly selected cells from all existing stacks are shown. In each case, the x-coordinate of the determined position of the cell nuclei (upper figure) or the y-coordinate (lower figure) as a function of the depth of invasion (referred to as Height) is shown.

Figure S4

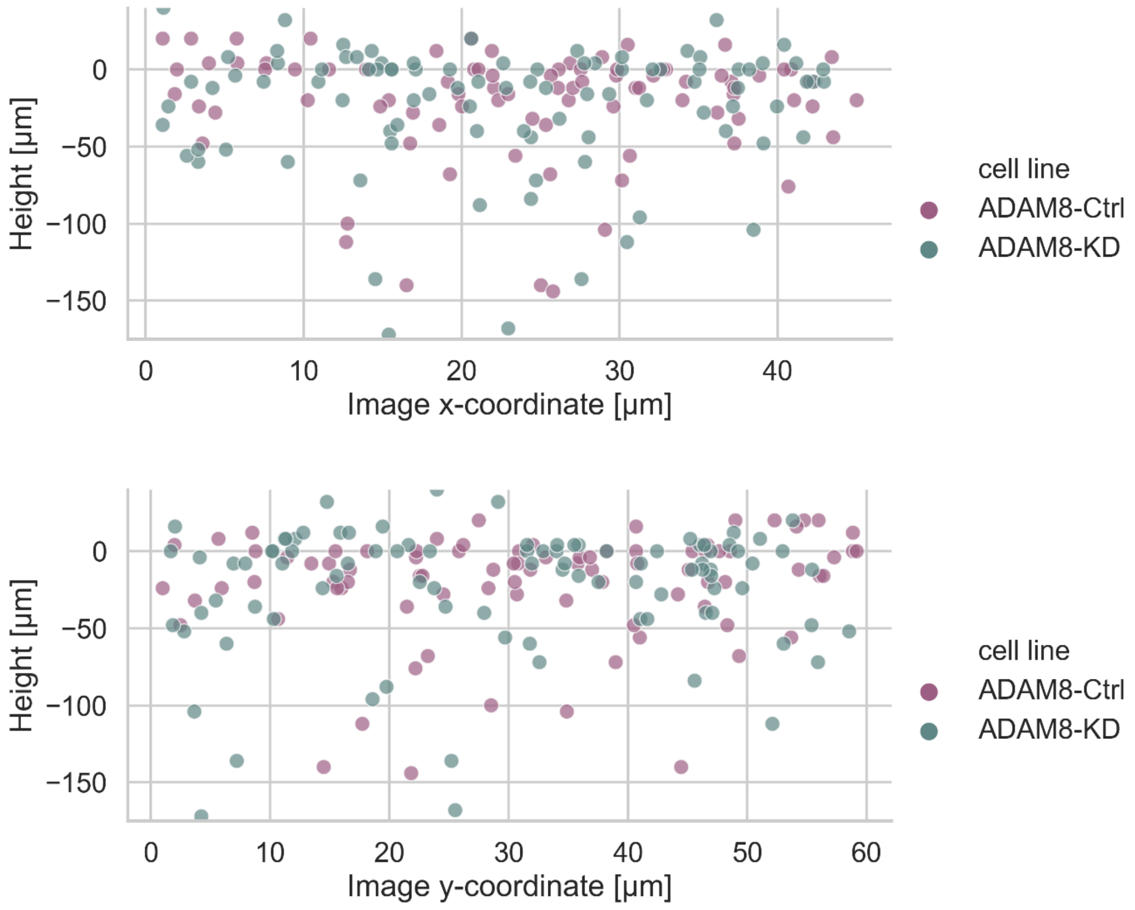

Figure S4: Exemplary comparison of the invasion of ADAM8-Ctrl cells and ADAM8-KD cells. 100 randomly selected cells from all existing stacks are shown. In each case, the x-coordinate of the determined position of the cell nuclei (upper figure) or the y-coordinate (lower figure) as a function of the depth of invasion (referred to as Height) is shown.

Figure S5

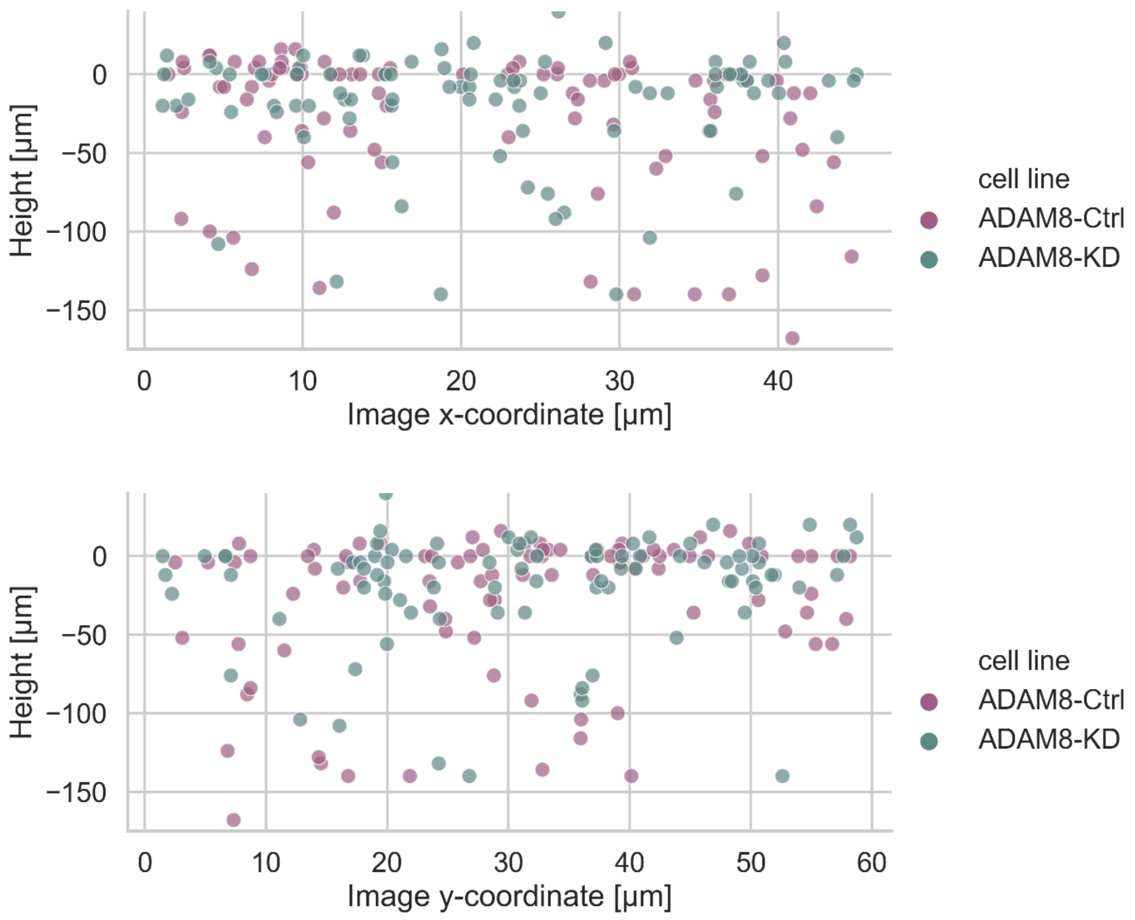

Figure S5: Exemplary comparison of the invasion of ADAM8-Ctrl cells in fibronectin-collagen matrices and ADAM8-KD cells in pure collagen matrices. 100 randomly selected cells from all existing stacks are shown. In each case, the x-coordinate of the determined position of the cell nuclei (upper figure) or the y-coordinate (lower figure) as a function of the depth of invasion (referred to as Height) is shown.

SV1 – 4: Videos of representative sample cells within collagen matrices with and without fibronectin content

SV1: ADAM8-Ctrl cell in a pure collagen matrix observed over 120 minutes

SV2: ADAM8-KD cells in a pure collagen matrix observed over 120 minutes

SV3: ADAM8-FN cells (ADAM8-Ctrl cells in contact with fibronectin) in a collagen-fibronectin matrix observed over 120 minutes

SV4: A8-KD-FN cells (ADAM8-KD cells in contact with fibronectin) in a collagen-fibronectin matrix observed over 120 minutes

Video description: SV1 and SV2 show the respective cells migrating in pure collagen matrices. Obviously, the ADAM8-KD cell (SV2) displaces the surrounding collagen fibers more intense than the ADAM8-Ctrl cell (SV1). In contact with fibronectin-collagen matrices the ADAM8-FN cell (ADAM8-Ctrl cell in contact with fibronectin, SV3) shows distinct higher displacements of the surrounding fiber meshwork than observable for the A8-KD-FN cell (ADAM8-KD cell in contact with fibronectin, SV4) and the cells within pure collagen matrices.
